# Mango Peel Fermented with Ethanol-Industry Yeast Waste and Urea Modulates In Vitro Rumen Fermentation, Nutrient Degradability, and Methane Concentration

**DOI:** 10.3390/ani16203237

**Published:** 2026-10-15

**Authors:** Rittikeard Prachumchai, Bhornchai Harakotr, Pipat Somparn, Mayulee Suwannakaew, Anusorn Cherdthong, Chanon Suntara, Saroat Rawdkuen

**Affiliations:** 1 Department of Agricultural Technology, Faculty of Science and Technology, Thammasat University, Pathum Thani 12120, Thailand; harakotr@tu.ac.th (B.H.); somparn@tu.ac.th (P.S.); 2 Faculty of Public Health, Thammasat University, Pathum Thani 12120, Thailand; mayulee.s@fph.tu.ac.th; 3 Department of Animal Science, Faculty of Agriculture, Khon Kaen University, Khon Kaen 40002, Thailand; anusornc@kku.ac.th (A.C.); chansun@kku.ac.th (C.S.); 4 Unit of Innovative Food Packaging and Biomaterials, School of Agro-Industry, Mae Fah Luang University, Chiang Rai 57100, Thailand; saroat@mfu.ac.th

**Keywords:** agroindustrial by-product, gas-production kinetics, *Mangifera indica*, methane mitigation, rumen protozoa, *Saccharomyces cerevisiae*

## Abstract

This study evaluated mango peel co-fermented with fresh yeast waste from the ethanol industry and urea (YUFMP) as a ruminant supplement using the in vitro gas-production technique. A 2 × 4 factorial arrangement in a completely randomised design was used, with two roughage-to-concentrate (R:C) ratios (40:60 and 60:40; rice straw as roughage) and four YUFMP levels (0, 2%, 4%, and 6% of substrate dry matter, DM). Co-fermentation raised the crude protein of mango peel from 4.39% to 17.00% DM and reduced its total phenolic content from 100.30 to 73.49 mg GAE/g extract. The 40:60 ratio gave higher in vitro degradability of DM, organic matter, neutral detergent fibre (IVNDFD), and true DM (IVTD) than the 60:40 ratio at 12 and 24 h (*p* < 0.01), and higher total volatile fatty acids (VFA) at 24 h (65.56 vs. 57.16 mmol/L; *p* < 0.01). YUFMP increased asymptotic gas production from 87.85 to 100.47–102.58 mL/0.5 g DM (*p* < 0.01) and cumulative gas production at 12–96 h (*p* < 0.05), with linear and quadratic trends. At 24 h, IVNDFD and IVTD were highest at 6% YUFMP (47.90 and 69.30%; *p* < 0.05). The protozoal population declined linearly with YUFMP (12.75 to 9.00 × 10^5^ cells/mL at 24 h; *p* < 0.01), as did CH_4_ concentration at 12 h (18.99 to 13.08%) and 24 h (23.21 to 14.93%; both *p* < 0.01), whereas absolute CH_4_ volume was unaffected (*p* > 0.05). YUFMP did not alter total VFA or VFA molar proportions at 24 h and reduced propionate linearly only at 12 h (*p* < 0.01), indicating that the lower CH_4_ concentration reflected dilution of CH_4_ within a larger volume of fermentation gas rather than a redirection of metabolic hydrogen towards propionate. Ruminal pH (6.87–7.04) and ammonia nitrogen were unaffected (*p* > 0.05). Inclusion at 2–4% DM is recommended for further in vivo evaluation.

## Simple Summary

Two waste streams are generated in large volumes across tropical Asia: peel left over when mangoes are processed, and the spent yeast sludge discharged by ethanol distilleries. Mango peel still contains sugars, fibre, and protective plant compounds, but almost no protein, whereas yeast sludge is rich in protein, yet difficult to store and handle. In this study, the two wastes were mixed and fermented together in sealed bags for 14 days, which produced a dry, protein-enriched supplement. The supplement was tested in the laboratory with rumen fluid from cattle so that several inclusion levels could be screened before any animal feeding trial. It was added at four levels to two diets differing in the proportion of roughage to concentrate. Adding the fermented product increased gas production and fibre digestion, progressively reduced the number of rumen protozoa, and lowered the proportion of methane in the fermentation gas while leaving rumen acidity and ammonia unchanged. The clearest benefits occurred at about 4% of diet dry matter. Combining two discarded materials can therefore create a feed ingredient that supports rumen function while reducing the methane concentration of the fermentation gas.

## 1. Introduction

The cost of imported protein and energy concentrates is a persistent constraint on ruminant production in tropical Asia, and recent geopolitical shocks have shown how volatile this cost can be. The 2022 war in Ukraine disrupted global grain, energy and fertiliser markets [1], and in Thailand, which relies heavily on imported feed ingredients, the prices of maize, wheat, and barley rose by 30.5–56.6% between 2021 and 2022 [2]. The 2026 conflict in the Middle East, which disrupted shipping through the Strait of Hormuz, has again raised energy and agricultural input costs and amplified price volatility in import-dependent regions, including Asia [3]. These pressures have renewed interest in agroindustrial by-products that are locally abundant, inexpensive, and currently discarded [4,5]. A second constraint must be addressed simultaneously: tropical ruminant diets rely on fibrous crop residues whose fermentation favours acetate production and methanogenesis, and the livestock sector contributes approximately 14.5% of anthropogenic greenhouse gas emissions, with enteric methane (CH_4_) its largest single component [6,7,8].

Mango (*Mangifera indica* L.) is among the most extensively processed tropical fruits. The peel accounts for 7–24% of fruit weight, and 40–60% of the fruit mass is discarded during processing [9]. The peel retains soluble sugars, pectin, and structural carbohydrate, together with a phenolic fraction that is more concentrated than in the pulp [4,9]. Mango by-products have partially replaced concentrate in dairy goat diets without impairing digestibility or milk yield [10], improved intake and growth of dairy calves when fed as peel silage [11], and shifted in vitro fermentation towards propionate while lowering CH_4_ [12]. This anti-methanogenic response has been linked directly to the phenolic fraction of the peel [13], consistent with the dose-dependent action of dietary phenolics on methanogenesis and rumen protozoa [14,15]. The principal limitation of mango peel is its very low crude protein (CP) content, which limits its value when it is fed with roughages that are equally deficient in protein, such as rice straw [4,16].

Spent yeast from the ethanol industry offers a complementary solution. Ethanol distilleries discharge large volumes of spent yeast sludge, a residue rich in *Saccharomyces cerevisiae* protein, nucleotides, minerals, and vitamins, but too wet to store or transport unless it is stabilised [17,18]. Yeast waste and yeast-fermented by-products have replaced soybean meal without impairing ruminal fermentation [18] and have improved fermentation, nutrient digestibility, nitrogen balance, and microbial protein synthesis in tropical cattle [19,20], while yeast cell-wall components may act as prebiotics [21] and yeast cultures can modify CH_4_ production in vitro [22]. Solid-state co-fermentation of a fibrous residue with this wet, protein-rich waste therefore stabilises the yeast waste while upgrading the fibrous residue, a principle already applied to citric acid waste, cassava pulp, and crop residues in Thailand [5,23,24]. Using citric waste fermented with yeast waste, Kanakai et al. [25] identified 4% of substrate dry matter (DM) as the upper safe inclusion level in high-concentrate diets, and several studies have shown that the response to such supplements depends on the roughage-to-concentrate (R:C) ratio of the basal diet [19,23,26,27].

Combining the two materials may also allow a functional interaction between them. Plant phenolics, particularly tannins, bind proteins through hydrogen bonding and hydrophobic interactions, forming complexes that are stable at ruminal pH, but dissociate under the acidic conditions of the abomasum [15,28]. At moderate concentrations, this binding slows ruminal proteolysis and deamination, reduces ammonia losses, and increases the flow of dietary protein to the small intestine [28,29]. At higher concentrations, free phenolics also complex with microbial enzymes and cell envelopes, depressing fibre degradation [14,29]. Mango peel contains xanthone and flavonol glycosides, notably mangiferin, together with hydrolysable tannins [9,30], whereas yeast waste supplies protein that may act as a binding sink for these compounds. Microbial fermentation can also hydrolyse or release bound phenolics [31], so co-fermentation may modify both the quantity and the reactivity of the phenolic fraction before the product reaches the rumen. The protein-to-phenolic balance of the fermented product may therefore determine whether its phenolics act as protein-protecting and anti-methanogenic agents or as inhibitors of fibre digestion.

To the best of our knowledge, mango peel co-fermented with ethanol-industry yeast waste has not been evaluated as a ruminant supplement, and the interaction between its inclusion level and the R:C ratio has not been described. The product was therefore designed so that yeast-derived protein and mango peel phenolics would interact during co-fermentation, moderating ruminal ammonia release while supplying fermentable substrate. It was hypothesised that the product would stimulate fermentation and fibre degradation, suppress the protozoal population, and reduce the CH_4_ concentration of the fermentation gas, and that these responses would depend on the R:C ratio. The objectives were to characterise the chemical and phytochemical composition of untreated and fermented mango peel and determine the effects of inclusion level (0, 2%, 4%, and 6% DM) at two R:C ratios (40:60 and 60:40) on gas-production kinetics, in vitro nutrient degradability, ruminal fermentation characteristics, protozoal population, and CH_4_ concentration and volume.

## 2. Materials and Methods

### 2.1. Ethics Statement

Rumen fluid was collected from three female non-fistulated Thai Brahman cows (*Bos indicus*; average body weight 450 ± 20 kg) maintained at the Faculty of Science and Technology, Thammasat University, Thailand. Donor animals were selected to be of similar age, body weight and health status to ensure uniformity of the rumen inoculum. All procedures involving animals were approved by the Institutional Animal Care and Use Committee of Thammasat University (protocol code IACUC-TU 011/2025, date of approval: 11 June 2025) and were conducted in accordance with the guidelines for the care and use of experimental animals.

### 2.2. Preparation of the Fermented Mango Peel Products

Mango peels were obtained from a commercial preserved-mango processing facility in Chachoengsao Province, Thailand. The peels originated from mature green fruits of the cultivars Chok Anan, Nam Dok Mai and Kaew Khiao, which are used for sweetened preserved-mango production. Raw materials were collected from three production lots and pooled to obtain a homogeneous substrate before fermentation. The pooled peel, including any residual flesh that remained attached after peeling, was washed to remove contaminants, dried at 60 °C to a DM content above 88%, ground to pass a 1 mm screen and stored in airtight containers until use. Fresh yeast waste sludge, the spent non-viable biomass of *Saccharomyces cerevisiae* discharged by the ethanol industry, was obtained with a moisture content of 85–92% and was analysed for DM immediately before use so that formulation could be carried out accurately on a DM basis. The yeast waste was not dried before use, in order to retain the physical condition in which it would be handled at industrial scale.

Fermentation mixtures were formulated on a total DM basis of 2.0 kg per batch, with yeast waste contributing 20% of total DM. Given a yeast waste DM of approximately 10%, this corresponded to 4.00 kg of fresh yeast waste (0.40 kg DM) mixed with 1.60 kg of dried mango peel DM. Because the yeast waste contributed approximately 3.60 kg of water, the moisture of the mixture was already close to the 60–65% target for solid-state fermentation and only 0–0.15 L of additional water was required. Molasses was added at 2% of total DM (40 g per batch) after dissolution in a small volume of water. Two products were prepared: mango peel fermented with yeast waste and molasses without urea (YFMP), and the same mixture with urea added at 1.5% of total DM (30 g per batch) before packing (YUFMP). Untreated dried mango peel (MP) served as the unfermented reference.

Ingredients were weighed, mixed until visually uniform and checked for even moisture distribution. The target was a material that held together when squeezed by hand without releasing free water. The mixtures were packed into airtight plastic bags, compressed to expel residual air, vacuum-sealed and fermented at ambient temperature (30–35 °C) for 14 days without aeration, following the general procedure used for yeast waste-fermented by-products by Kanakai et al. [25] and Suriyapha et al. [5]. After fermentation, each bag was opened, a subsample was taken for DM determination, and the material was dried at 60 °C to below 10% moisture and ground to a uniform particle size before storage in moisture-proof containers. The DM content of the fermented YUFMP before drying was 33.67%. Only YUFMP was used as the supplement in the in vitro treatments. MP and YFMP were characterised chemically for comparison (Table 1).

### 2.3. Experimental Design and Treatments

The experiment was arranged as a 2 × 4 factorial in a completely randomised design (CRD). The first factor was the R:C ratio of the basal substrate (40:60 and 60:40, DM basis), with rice straw as the roughage source. Rice straw was selected as the roughage because it is the most abundant crop residue and the principal basal roughage fed to ruminants by smallholder farmers in Thailand and much of tropical Asia, and its low CP content (4.81% DM; Table 1) represents the protein-deficient basal diet that YUFMP is intended to complement [4,16]. The second factor was the inclusion level of YUFMP, applied as a top-dressed supplement at 0, 2%, 4%, and 6% of substrate DM. Top-dressing, rather than substitution for part of the basal substrate, was chosen to mimic the intended on-farm use of YUFMP as a supplement added to an existing ration so that the composition of the basal substrate remained identical across inclusion levels. Only YUFMP, the product in its final intended form, was incubated. MP and YFMP were characterised chemically to document the compositional changes brought about by co-fermentation and the addition of urea. YUFMP was selected for incubation because it had the highest CP concentration of the three products (17.00% DM) and represents the formulation intended for practical use as a combined source of nitrogen and fermentable substrate. The experiment was therefore designed as a dose–response evaluation of this product at two R:C ratios rather than as a comparison among products. The eight treatments were: T1 = 40:60 with 0% YUFMP; T2 = 40:60 with 2%; T3 = 40:60 with 4%; T4 = 40:60 with 6%; T5 = 60:40 with 0%; T6 = 60:40 with 2%; T7 = 60:40 with 4%; and T8 = 60:40 with 6%. The basal substrate supplied 0.5 g DM per incubation vial, and YUFMP was weighed in addition to the basal substrate at the stated percentage of substrate DM. Accordingly, YUFMP supplied 0.01, 0.02 and 0.03 g DM per vial at the 2%, 4%, and 6% levels, so that the total DM incubated was 0.51, 0.52 and 0.53 g, respectively. The calculated chemical composition of the eight incubated substrates, derived from the analysed composition of each component (Table 1) and the amounts incubated, is presented in Table S1. The ingredient composition of the concentrate and the chemical composition of all feed components are presented in Table 1.

### 2.4. Chemical and Phytochemical Analysis

Feed components and mango peel products were dried at 60 °C for 48 h and ground to pass a 1 mm screen. Dry matter (method 930.15) and ash (method 942.05) were determined according to AOAC [32], with organic matter (OM) calculated by difference. Crude protein was determined by the Kjeldahl method (method 976.05; N × 6.25) [32]. Neutral detergent fibre (NDF) and acid detergent fibre (ADF) were determined sequentially with heat-stable alpha-amylase and sodium sulphite using an ANKOM 200 Fibre Analyser (ANKOM Technology, Macedon, NY, USA) according to Van Soest et al. [33] and are expressed on an ash-inclusive basis. All chemical and phytochemical analyses were performed in triplicate (*n* = 3), and values are reported as means.

For phytochemical analysis, 5 g of ground sample was extracted three times with 95% ethanol at a 1:3 (*w*/*v*) ratio over 72 h and filtered; the pooled extracts were dried in a hot-air oven at 50 °C for 72 h and the dried extracts stored at −20 °C until analysis, following Harakotr et al. [34] and Jirakiattikul et al. [35]. Total phenolic content (TPC) was determined by the Folin–Ciocâlteu colorimetric method [36] and total flavonoid content (TFC) by the method of Kubola et al. [37], with absorbance read at 765 and 510 nm, respectively, in a microplate reader. Results are expressed as mg gallic acid equivalent (GAE) and mg quercetin equivalent (QE) per gram of dried extract. Antioxidant activity was measured by the 2,2-azino-bis(3-ethylbenzothiazoline-6-sulfonic acid) (ABTS) radical cation decolorisation assay of Re et al. [38] and the 2,2-diphenyl-1-picrylhydrazyl (DPPH) radical scavenging assay of Brand-Williams et al. [39], with absorbance read at 734 and 520 nm, respectively. Radical scavenging activity was calculated as [(A_control_ − A_sample_)/A_control_] × 100, where A_control_ and A_sample_ are the absorbance of the radical solution without and with the extract, and expressed as a percentage. Because phytochemical results are expressed per gram of dried extract, they allow direct comparison among the mango peel products, but are not intended to represent concentrations on a feed DM basis.

### 2.5. Rumen Fluid Preparation and In Vitro Incubation

Rumen fluid was collected from the three non-fistulated Thai Brahman donor cows described in Section 2.1, which were fed rice straw ad libitum with concentrate at 1% of body weight. Rumen contents were collected before the morning feeding via oral stomach tube: a flexible polyethylene tube was inserted transorally through a speculum into the rumen, and fluid was withdrawn by gentle vacuum suction using a hand-operated pump into a pre-warmed collection vessel [25]. To minimise salivary contamination, the first portion of aspirate was discarded, and the pH of the rumen fluid measured immediately after collection (6.8–6.9) was within the normal range for pre-feeding ruminal fluid, indicating negligible salivary contamination. The fluid was immediately strained through four layers of cheesecloth and transported to the laboratory in pre-warmed insulated flasks under continuous CO_2_ flushing. Fluid from the three donors was pooled and held at 39 °C under CO_2_. Because it was pooled before incubation, donor animals were not used as an experimental unit. Buffered rumen fluid was prepared by mixing rumen fluid with CO_2_-saturated McDougall’s artificial saliva [40] at a ratio of 1:2 (*v*/*v*), with pH adjusted to 6.8 ± 0.1.

Substrates were weighed into 50 mL serum vials, flushed with CO_2_, sealed with rubber stoppers and aluminium caps, and incubated with 40 mL of buffered rumen fluid at 39 °C without agitation using the in vitro gas-production technique [41,42]. This corresponded to an inoculum-to-substrate ratio of 80 mL of buffered rumen fluid (approximately 26.7 mL of rumen fluid) per gram of basal substrate DM. Three independent sets of vials were prepared from the same batch of buffered rumen fluid: Set A for cumulative gas production (three vials per treatment plus three blanks containing buffered rumen fluid only), Set B for CH_4_ and fermentation characteristics (CH_4_, pH, NH_3_-N, VFAs and protozoa; two vials per treatment per time point at 12 and 24 h), and Set C for in vitro nutrient degradability (two vials per treatment per time point at 12 and 24 h). The vials of all three sets were handled identically: at each gas-reading time (0.5, 1, 2, 4, 6, 8 and 12 h, and 24 h for the 24 h vials), the gas accumulated in the headspace was measured and released through the stopper with a syringe needle so that the headspace returned to atmospheric pressure and pressure could build up only between consecutive readings [41,43]. Blank vials were included in each set to correct for gas and residue originating from the inoculum, and gas-production and degradability values are reported on a blank-corrected basis.

Gas volume was measured manually by reading the displacement of a calibrated 20 mL graduated glass syringe at 0.5, 1, 2, 4, 6, 8, 12, 24, 36, 48, 72 and 96 h after inoculation, following the technique of Menke and Steingass [41]. Cumulative gas-production data were fitted by non-linear least squares regression to the exponential model with a discrete lag term described by Schofield [44], a modification of the model of Ørskov and McDonald [45]: *V*(*t*) = *b* × [1 − exp^(−c(t − L))^], where *V*(*t*) is cumulative gas volume at time t, *b* is asymptotic gas production corresponding to complete substrate fermentation (mL/0.5 g DM), c is the fractional rate of gas production (h^−1^) and *L* is the discrete lag time (h). Vessels that failed to establish fermentation were excluded according to the criterion described in Section 2.9.

### 2.6. Rumen Fermentation Characteristics

At 12 and 24 h, after the headspace gas had been sampled for CH_4_ analysis (Section 2.7), the Set B vials were removed from the incubator and their contents collected. Ruminal pH was measured immediately with a calibrated glass electrode. Samples were centrifuged at 3000× *g* for 15 min at 4 °C and the supernatant used for NH_3_-N determination; a separate 1 mL aliquot of fresh fluid was retained before centrifugation for protozoal enumeration. Ruminal NH_3_-N was determined colorimetrically by the phenol–hypochlorite method [46] against a standard curve prepared from ammonium sulphate, with absorbance read at 630 nm, and is expressed as mg/dL. Protozoa were enumerated by the methyl-green–formalin–saline staining method of Galyean [47]: a 1 mL aliquot of fresh rumen fluid was fixed in the staining solution (1:1, *v*/*v*) and total protozoa counted under a light microscope with a haemocytometer at ×100 magnification. Results are expressed as ×10^5^ cells/mL.

Volatile fatty acid (VFA) concentrations were determined by high-performance liquid chromatography following Samuel et al. [48]. Supernatant from the Set B vials collected at 12 and 24 h was deproteinised with metaphosphoric acid (25%, *w*/*v*) and centrifuged at 12,000× *g* for 15 min at 4 °C. The clarified supernatant was filtered through a 0.22-micrometre membrane and injected onto an Aminex HPX-87H organic acid column (Bio-Rad Laboratories, Hercules, CA, USA) at 60 °C, with 0.005 M H_2_SO_4_ as mobile phase at 0.6 mL/min, refractive index detection and crotonic acid as internal standard. Each sample was injected in duplicate and the mean of the two injections was used. Total VFAs are expressed as mmol/L, and acetate, propionate, and butyrate are expressed as mol/100 mol of their sum.

### 2.7. Methane Determination

Methane was measured directly by gas chromatography at 12 and 24 h in the headspace of the Set B vials (two per treatment per time point), immediately before the vials were opened for sampling. At each time point a 10 mL headspace gas sample was withdrawn with a gas-tight syringe and injected into a gas chromatograph fitted with a flame ionisation detector (Shimadzu Corporation, Kyoto, Japan). Separation used a stainless-steel packed column (Porapak Q, 80/100 mesh; 2 m × 3 mm) with nitrogen carrier gas at 30 mL/min; column, injector and detector temperatures were 50, 100 and 150 °C. Methane concentration was quantified against a certified CH_4_ standard gas by external calibration and is expressed as a percentage of headspace gas (% *v*/*v*). Methane volume (mL) was calculated for each Set B vial as the product of its CH_4_ concentration and the blank-corrected cumulative gas volume, at the same time point, of the Set A vessel with which it was paired, and treatment means were computed from these individual values. Methane production was additionally expressed per gram of basal substrate DM (0.5 g).

### 2.8. In Vitro Nutrient Degradability

In vitro dry matter degradability (IVDMD) and organic matter degradability (IVOMD) were determined on the Set C vials at 12 and 24 h using a filtration and ignition procedure adapted from the two-stage technique of Tilley and Terry [49]. At the end of each incubation, the vial contents were quantitatively filtered through pre-weighed Whatman filter paper under vacuum, rinsed with distilled water, dried at 105 °C for 24 h and weighed to determine the undegraded DM residue. The dried residue and filter paper were then ignited at 550 °C for 3 h to determine residue ash, from which undegraded OM was obtained by difference.

In vitro neutral detergent fibre degradability (IVNDFD) and true degradability (IVTD) were determined on separate vials using pre-weighed nylon filter bags (ANKOM Technology, Macedon, NY, USA). At the end of incubation, the bags were removed, rinsed with distilled water and refluxed in neutral detergent solution [33] to remove soluble fermentation products and microbial biomass, then rinsed with distilled water and acetone, dried at 105 °C for 24 h and weighed to obtain the neutral-detergent-insoluble residue.

Degradability coefficients were calculated as follows: IVDMD (%) = [(DM incubated − undegraded DM residue)/DM incubated] × 100; IVOMD (%) = [(OM incubated − undegraded OM residue)/OM incubated] × 100; IVNDFD (%) = [(NDF incubated − neutral-detergent-insoluble residue)/NDF incubated] × 100; and IVTD (%) = [(DM incubated − neutral-detergent-insoluble residue)/DM incubated] × 100. The NDF and OM contents of each substrate were calculated as the DM-weighted mean of the rice straw, concentrate and YUFMP fractions actually weighed into each vial.

### 2.9. Statistical Analysis

Data were analysed with the MIXED procedure of SAS (version 9.4; SAS Institute Inc., Cary, NC, USA) as a 2 × 4 factorial arrangement in a CRD, following standard procedures for factorial designs [50,51]. The model included the R:C ratio, the YUFMP inclusion level and their interaction as fixed effects, and each incubation time was analysed separately. Treatment-combination means were separated with Duncan’s multiple range test only when the R:C ratio × YUFMP interaction was significant. When the interaction was not significant, the R:C ratio and YUFMP level main-effect means were separated instead. The response to inclusion level was further partitioned into orthogonal polynomial contrasts (linear, quadratic and cubic), which were orthogonal because the four levels were equally spaced. Significance was declared at *p* < 0.05. In the tables, lowercase superscripts on the eight treatment means are shown only when the R:C ratio × YUFMP interaction was significant. Otherwise, lowercase and uppercase superscripts on the main-effect means (marginal means of the treatment cells) denote differences between R:C ratios and among YUFMP levels, respectively, provided that the corresponding main effect was significant. Cubic contrasts were not significant for any variable (*p* > 0.05) and are therefore not shown. Relationships of CH_4_ concentration and volume with IVNDFD, cumulative gas production, protozoal count and the A:P ratio were examined by Pearson correlation of the treatment means (*n* = 8) using the CORR procedure of SAS.

Each incubation vial served as the experimental unit, with three vials per treatment for the gas-production measurements and two vials per treatment per time point for the fermentation and degradability measurements. Vessels that failed to establish fermentation, defined a priori as a cumulative gas profile that did not converge on the fermentation model or that remained below 20% of the treatment mean, were excluded from the gas-production dataset. This criterion applied to three vessels. For the gas variables, the SEM reported is the largest standard error among the treatment means in each column, and Duncan critical ranges were based on the harmonic mean sample size.

## 3. Results

### 3.1. Chemical Composition of the Feed Components and Mango Peel Products

The ingredient composition of the concentrate and the chemical composition of the feed components and mango peel products are presented in Table 1. Co-fermentation with yeast waste markedly altered the composition of the mango peel. Untreated mango peel (MP) contained 4.39% CP, 38.01% NDF and 13.72% ADF on a DM basis, with 5.85% ash. Fermentation with yeast waste alone (YFMP) raised CP to 13.20% DM, and the addition of urea at 1.5% of DM (YUFMP) raised it further to 17.00% DM, approximately 3.9 times the CP of the untreated peel. Both fermented products had higher fibre and ash concentrations than the untreated peel (NDF 44.76 and 44.02%, ADF 22.69 and 23.56%, ash 11.22 and 10.56% DM for YFMP and YUFMP, respectively). The CP content of YUFMP (17.00% DM) exceeded that of the concentrate (15.53% DM), whereas the rice straw used as roughage contained 4.81% CP, 75.95% NDF and 43.50% ADF on a DM basis.

The phytochemical composition of the mango peel products is also presented in Table 1. Untreated MP contained 100.30 mg GAE/g extract of total phenolics and 110.14 mg QE/g extract of total flavonoids. Co-fermentation reduced TPC by 24.5% in YFMP (75.73 mg GAE/g extract) and by 26.7% in YUFMP (73.49 mg GAE/g extract), whereas TFC increased slightly to 120.38 and 120.85 mg QE/g extract, respectively. The two antioxidant assays responded in opposite directions: DPPH radical scavenging activity increased from 35.72% in MP to 37.51% in YFMP and 40.52% in YUFMP, whereas ABTS radical scavenging activity decreased from 43.78% to 29.29 and 25.26%, respectively.

### 3.2. In Vitro Nutrient Degradability

**Figure 1:**
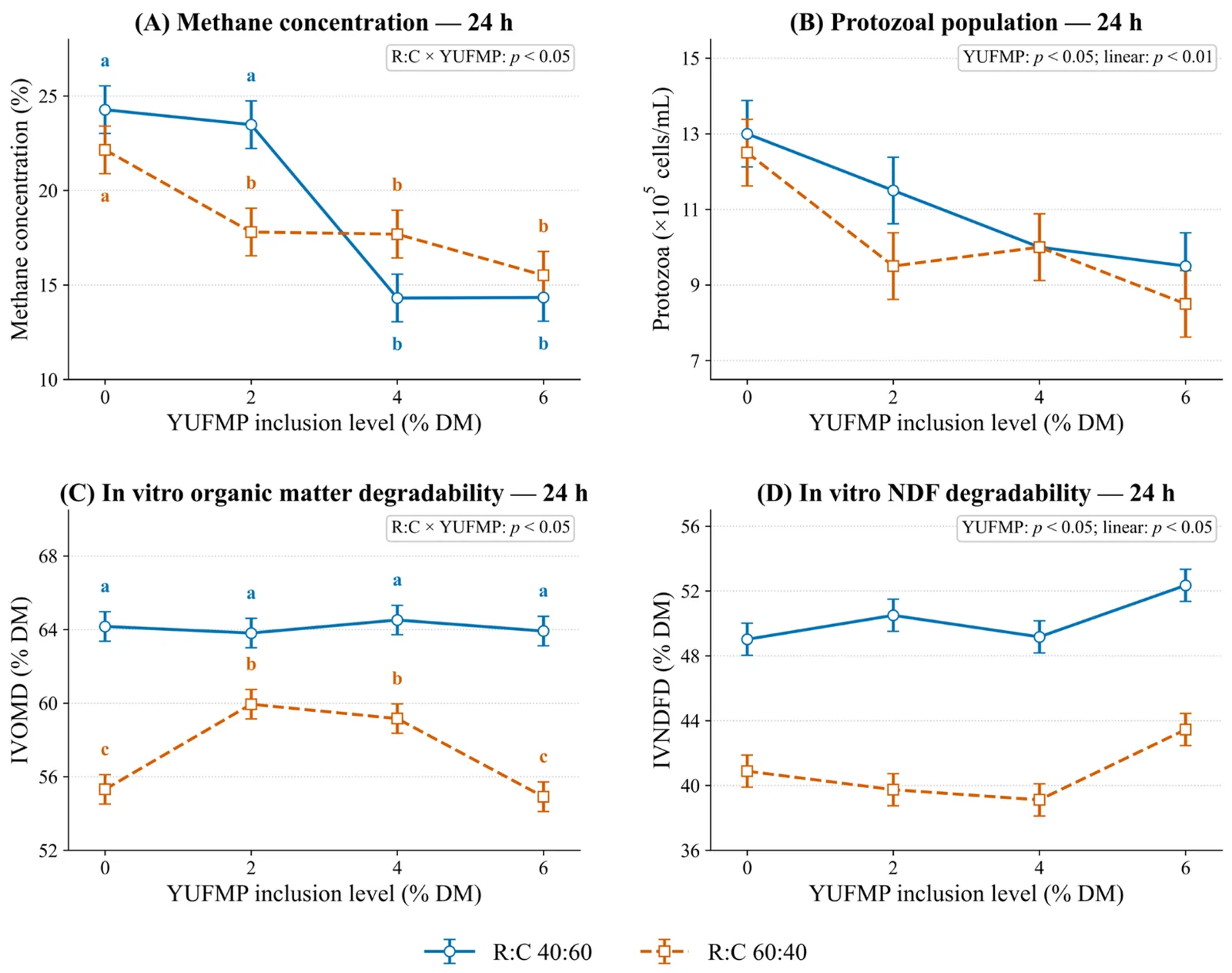
Dose–response effects of mango peel co-fermented with yeast waste and urea (YUFMP) inclusion under two roughage-to-concentrate (R:C) ratios on (**A**) methane concentration, (**B**) protozoal population, (**C**) in vitro organic matter degradability (IVOMD), and (**D**) in vitro neutral detergent fibre degradability (IVNDFD) after 24 h of incubation. Error bars represent the standard error of the mean. Different lowercase letters (a–c) in panels A and C indicate differences among treatment means (*p* < 0.05) and are shown only where the R:C ratio × YUFMP interaction was significant.

In vitro nutrient degradability is presented in Table 2, and the dose–response of IVOMD and IVNDFD at 24 h is illustrated in Figure 1C,D. The R:C ratio affected IVDMD, IVOMD, IVNDFD and IVTD at both incubation times (all *p* < 0.01), with the 40:60 ratio consistently higher than the 60:40 ratio (for example, IVDMD 60.62 vs. 53.76% and IVTD 72.19 vs. 63.14% at 24 h). At 12 h the R:C ratio × YUFMP interaction was significant for all four variables (*p* < 0.01). Within the 40:60 ratio, T1 had the highest IVDMD (45.59%) and T3 the lowest (41.80%), whereas within the 60:40 ratio IVDMD was lowest in T5 and T6 (34.74 and 33.65%) and increased to 40.30% in T8. IVOMD followed the same pattern (highest in T1, 46.58%; lowest in T6, 35.27%), and IVTD ranged from 64.11% in T1 to 52.75% in T5.

At 24 h, the interaction remained significant for IVOMD (*p* < 0.05), but not for IVDMD (*p* = 0.0706), IVNDFD (*p* = 0.5769), or IVTD (*p* = 0.5430). For IVOMD at 24 h, T1, T2, T3 and T4 (63.81–64.52%) did not differ from each other and all exceeded T6 and T7 (59.94 and 59.16%), which in turn exceeded T5 and T8 (55.31 and 54.91%). The YUFMP level affected IVDMD, IVOMD, IVNDFD and IVTD at 24 h (*p* < 0.05). Averaged over the R:C ratios, IVDMD was highest at 2% YUFMP (58.62%) and lowest at 6% (55.90%), whereas IVNDFD and IVTD were highest at 6% YUFMP (47.90 and 69.30%, respectively) and did not differ among the 0, 2%, and 4% levels (44.14–45.12% and 66.92–67.33%). Quadratic contrasts were significant for all four degradability variables at both incubation times (*p* < 0.05), and linear contrasts were significant for IVNDFD and IVTD at 24 h (*p* < 0.05 and *p* < 0.01).

### 3.3. Ruminal pH, Ammonia Nitrogen and Protozoal Population

Ruminal pH, NH_3_-N concentration and protozoal population are presented in Table 3. Ruminal pH remained within a narrow range at 12 h (7.00–7.04) and 24 h (6.87–7.02) and was not affected by the R:C ratio, the YUFMP level or their interaction (all *p* > 0.05). Ammonia nitrogen ranged from 11.15 to 13.23 mg/dL at 12 h and from 8.79 to 11.98 mg/dL at 24 h and was likewise unaffected by treatment (all *p* > 0.05).

The protozoal population (Figure 1B) was not affected by the R:C ratio or the interaction at either time point (*p* > 0.05). The YUFMP level did not significantly affect the protozoal population at 12 h (*p* = 0.0693), although a significant linear decline was detected (*p* < 0.05; 12.50 to 9.25 × 10^5^ cells/mL from 0 to 6% YUFMP). At 24 h the YUFMP level effect was significant (*p* < 0.05) with a significant linear trend (*p* < 0.01): the 0% level had the highest count (12.75 × 10^5^ cells/mL), which differed from the 2%, 4%, and 6% levels (10.50, 10.00 and 9.00 × 10^5^ cells/mL), representing a 29.4% reduction at the 6% level relative to the unsupplemented control.

### 3.4. Methane Concentration and Production

Methane data are presented in Table 4, and the response of CH_4_ concentration at 24 h is illustrated in Figure 1A. At 12 h, CH_4_ concentration was affected by the R:C ratio (*p* < 0.01) and the YUFMP level (*p* < 0.05) but not by their interaction (*p* = 0.5981). The 40:60 ratio had a higher CH_4_ concentration than the 60:40 ratio (18.84 vs. 13.79%), and CH_4_ concentration declined linearly with YUFMP inclusion (*p* < 0.01) from 18.99% at 0% to 13.08% at 6%, a reduction of 31.1%. At 24 h, CH_4_ concentration was affected by the YUFMP level (*p* < 0.01) and by the R:C ratio × YUFMP interaction (*p* < 0.05) but not by the R:C ratio alone (*p* = 0.3883). Within the 40:60 ratio, T1 and T2 (24.27 and 23.48%) had higher CH_4_ concentrations than T3 and T4 (14.31 and 14.34%); within the 60:40 ratio, T5 (22.15%) exceeded T6, T7 and T8 (15.51–17.80%). The linear contrast across YUFMP levels was significant at 24 h (*p* < 0.01; 23.21 to 14.93%).

In contrast, CH_4_ volume expressed either in absolute terms (mL) or per gram of basal substrate DM was not affected by the R:C ratio, the YUFMP level or their interaction at either incubation time (all *p* > 0.05). Values ranged from 2.18 to 4.39 mL (4.36–8.79 mL/g DM) at 12 h and from 5.25 to 8.00 mL (10.51–16.00 mL/g DM) at 24 h. The linear contrast for CH_4_ volume at 24 h approached, but did not reach significance (*p* = 0.0810). Pearson correlation coefficients between CH_4_ and IVNDFD, cumulative gas production, protozoal count and the A:P ratio are presented in Table S2. CH_4_ concentration at 24 h was negatively correlated with cumulative gas production (r = −0.83, *p* = 0.011) and positively correlated with protozoal count (r = 0.88, *p* = 0.004), whereas none of the other correlations was significant (*p* > 0.05).

### 3.5. Gas-Production Kinetics and Cumulative Gas Production

**Figure 2:**
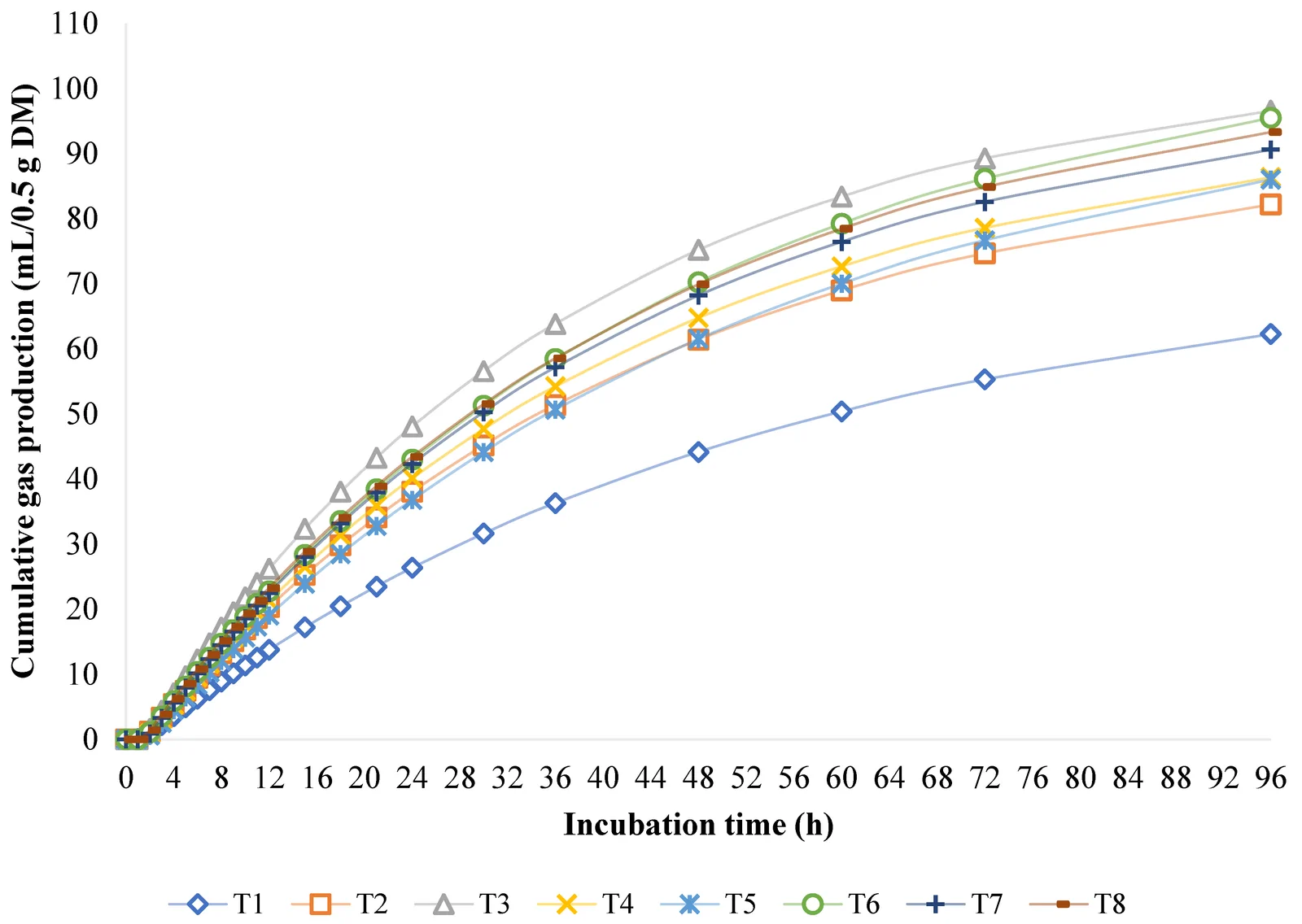
Fitted cumulative gas production of substrates containing 0, 2, 4, or 6% mango peel co-fermented with yeast waste and urea (YUFMP) under roughage-to-concentrate (R:C) ratios of 40:60 and 60:40 during 96 h of in vitro incubation.

Gas-production kinetics and cumulative gas production are presented in Table 5, and the fitted gas-production curves are shown in Figure 2. Asymptotic gas production (b) was affected by the R:C ratio (*p* < 0.01), the YUFMP level (*p* < 0.01) and their interaction (*p* < 0.01). The lowest b value was recorded for T1 (R:C 40:60 without YUFMP; 74.15 mL/0.5 g DM), which differed from all other treatments, whereas T6 had the highest value (109.47 mL/0.5 g DM); T3, T5, T7 and T8 (100.67–104.50 mL/0.5 g DM) did not differ from T6. Averaged over the R:C ratios, b increased from 87.85 mL/0.5 g DM at 0% YUFMP to 100.87, 102.58 and 100.47 mL/0.5 g DM at 2%, 4%, and 6% YUFMP, with significant linear and quadratic components (both *p* < 0.01). The fractional rate of gas production (c) was not significantly affected by the R:C ratio (*p* = 0.4934) or the YUFMP level (*p* = 0.0526), although a significant linear trend was detected across YUFMP levels (*p* < 0.05; 0.0200 to 0.0259 h^−1^ from 0 to 4% YUFMP). Lag time was not affected by any source of variation (1.41–1.82 h; *p* > 0.05).

Cumulative gas production at 6 h was not affected by treatment (*p* > 0.05). At 12 and 24 h, it was affected by the YUFMP level (*p* < 0.05) but not by the R:C ratio or the interaction; the 0% level produced the least gas (14.55 and 31.55 mL/0.5 g DM at 12 and 24 h), whereas the 2–6% levels produced 21.85–24.27 mL/0.5 g DM at 12 h and 38.93–44.82 mL/0.5 g DM at 24 h, with significant linear and quadratic trends (*p* < 0.05). At 48 and 96 h, cumulative gas production was affected by the R:C ratio (*p* < 0.05 and *p* < 0.01), the YUFMP level (both *p* < 0.01) and the interaction (both *p* < 0.05). Within the 40:60 ratio, T3 (4% YUFMP) produced the most gas at 48 h (79.10 mL/0.5 g DM) and 96 h (94.50 mL/0.5 g DM) and T1 the least (46.20 and 61.30 mL/0.5 g DM); within the 60:40 ratio the differences among YUFMP levels were smaller, and T6 reached 93.70 mL/0.5 g DM at 96 h.

### 3.6. Volatile Fatty Acid Concentrations

Total VFA concentration and the molar proportions of individual VFAs are presented in Table 6. At 12 h, total VFAs (44.11–50.63 mmol/L), acetate and butyrate were not affected by the R:C ratio, the YUFMP level or their interaction (*p* > 0.05). Propionate was affected by the YUFMP level (*p* < 0.05) and by the R:C ratio × YUFMP interaction (*p* < 0.05), with a significant linear decline across YUFMP levels (*p* < 0.01). Within the 40:60 ratio, T1–T4 (21.56–23.14 mol/100 mol) did not differ, whereas within the 60:40 ratio propionate decreased from 23.13 mol/100 mol in T5 to 20.35 mol/100 mol in T7, the lowest value among all treatments; T8 (21.32 mol/100 mol) was also lower than T1, T3 and T5. Correspondingly, the A:P ratio increased linearly with YUFMP inclusion at 12 h (*p* < 0.05; from 2.46 at 0% to 2.70 at 6% YUFMP), although the overall YUFMP effect was not significant (*p* = 0.0971).

At 24 h, the R:C ratio affected total VFAs, acetate and butyrate (all *p* < 0.01). The 40:60 ratio produced a higher total VFA concentration than the 60:40 ratio (65.56 vs. 57.16 mmol/L) and a higher molar proportion of butyrate (16.15 vs. 11.72 mol/100 mol), whereas the 60:40 ratio had a higher proportion of acetate (63.12 vs. 59.39 mol/100 mol). Propionate (23.43–26.39 mol/100 mol) and the A:P ratio (2.26–2.69) were not affected by the R:C ratio. YUFMP inclusion did not affect total VFAs, the individual VFA proportions or the A:P ratio at 24 h, and no interaction was detected (all *p* > 0.05); butyrate showed only a tendency to decline linearly with YUFMP inclusion (*p* = 0.0955; 15.20 to 13.24 mol/100 mol).

## 4. Discussion

### 4.1. Upgrading of Mango Peel by Co-Fermentation with Yeast Waste

Co-fermentation with fresh yeast waste transformed a fruit-processing residue of negligible protein value into an ingredient with a CP concentration comparable to a commercial concentrate. The increase from 4.39% CP in untreated peel to 13.20% in YFMP and 17.00% in YUFMP is consistent with the arithmetic of the formulation: yeast waste supplied 20% of the mixture DM, and spent *S. cerevisiae* biomass typically contains 40–50% CP [17,18]. The remaining enrichment is attributable partly to the loss of readily fermentable carbohydrate during solid-state fermentation, which is consistent with the concomitant rise in NDF (38.01% to 44.02% DM); the rise in ash (5.85% to 10.56% DM) may also reflect the mineral contribution of the yeast waste. On the basis of the DM content calculated for the mixture at packing (approximately 35%) and that measured after fermentation (33.67%), and assuming that water was retained in the vacuum-sealed bags, DM loss during fermentation was estimated at approximately 6% (range 2–8%, depending on the DM content of the starting materials), indicating that most of the substrate DM was recovered. Comparable enrichment has been documented repeatedly for this class of product. Suriyapha et al. [5] obtained 535 g/kg DM CP when citric waste was fermented with yeast waste, and Kanakai et al. [25] reported 590 g/kg DM CP for the pelleted equivalent. Polyorach et al. [19] and Sommai et al. [20] achieved similar upgrading of cassava-based substrates. The urea contribution can be accounted for explicitly. At 1.5% of DM and a crude protein equivalent of approximately 292%, urea supplied about 4.4 CP percentage points. The observed YUFMP value of 17.00% CP is therefore close to the 17.6% predicted from YFMP plus urea, and the small shortfall is consistent with modest ammonia volatilisation during fermentation and oven-drying. On this basis, roughly a quarter of the crude protein of YUFMP is non-protein nitrogen and the remainder is yeast-derived true protein and residual substrate nitrogen. From a practical standpoint, YUFMP (17.00% CP) could be incorporated into rice straw-based diets (4.81% CP) as a combined source of fermentable nitrogen and energy rather than as a bulk filler [16,24].

Co-fermentation also modified the phenolic fraction on which the anti-methanogenic rationale of the product depends. Total extractable phenolics declined by about a quarter, whereas total flavonoids increased slightly. Several processes could account for the decline in extractable phenolics during solid-state fermentation with a protein-rich partner: complexation of phenolics with yeast protein, which renders them less extractable by aqueous ethanol [28,29]; microbial hydrolysis or transformation of phenolic compounds [31]; and dilution of the peel-derived fraction by yeast solids, which contributed 20% of the mixture DM. The present data cannot separate these processes. Complexation is however the only one expected to alter the ruminal behaviour of the product, and it therefore remains a hypothesis to be tested directly. The slight rise in flavonoids is consistent with the release of bound flavonoids reported for fermented plant materials [31]. The divergence between the DPPH and ABTS assays is not contradictory. The two radicals differ in their reactivity towards hydrophilic and lipophilic antioxidants, so a change in the composition rather than the quantity of the phenolic pool can move the two indices in opposite directions [31,38,39]. Importantly, YUFMP retained a substantial phenolic and flavonoid fraction (73.49 mg GAE/g and 120.85 mg QE/g extract), which may contribute to the antiprotozoal response discussed below.

### 4.2. Stimulation of Gas Production and the Optimal Inclusion Level

The most consistent effect of YUFMP inclusion was the stimulation of fermentative activity. Asymptotic gas production increased by 15% on average, and cumulative gas at 24 h rose from 31.55 mL/0.5 g DM in the unsupplemented substrate to 44.82 mL/0.5 g DM at 4% YUFMP, a 42% increase. Because YUFMP was top-dressed, part of this response could in principle reflect the additional substrate supplied. The magnitude of the response argues against this as the main explanation: inclusion at 4% increased the DM incubated by only 4%, whereas cumulative gas increased by 42% at 24 h and by 67% at 12 h (from 14.55 to 24.27 mL/0.5 g DM), approximately ten times the increase that the added substrate mass alone could support. Top-dressing also altered the composition of the incubated substrate only marginally: at the 6% level, YUFMP raised the calculated CP concentration of the substrate by 0.33 and 0.45 percentage units in the 40:60 and 60:40 ratios, respectively, and lowered NDF by 0.7 and 1.1 percentage units (Table S1). Water-soluble carbohydrate and rumen-degradable protein were not determined; however, given the small mass of YUFMP added and the loss of readily fermentable carbohydrate during solid-state fermentation (Section 4.1), changes in these fractions are unlikely to account for the magnitude of the gas response, although a contribution of the readily available nitrogen supplied by YUFMP cannot be excluded. Two further features of the response deserve emphasis. First, the fractional rate (c) showed only a linear trend and lag time was unaffected, indicating that YUFMP increased the extent rather than the initial rate of fermentation, a pattern more consistent with an improved supply of fermentable nitrogen and substrate to the microbial population than with accelerated microbial attachment [25,44]. Second, the response was quadratic at every cumulative time point from 12 h onwards (*p* < 0.05), with the highest values at 4% YUFMP in the 40:60 ratio and no further increase at 6%. The most probable explanation for this plateau is the combined effect of phenolic accumulation and increased fibre load. Mango peel phenolics are concentrated in the peel fraction [4,9], and Geerkens et al. [13] showed that processed mango peel and its phenolic extract progressively suppressed in vitro gas production as dose increased, a pattern consistent with the dose-dependent inhibition described for dietary phenolics generally [14,15]. In parallel, each increment of YUFMP added fibre as well as nutrients, and Kanakai et al. [25] attributed the decline they observed at 6% inclusion to exactly this increase in total fibre mass physically limiting microbial access to fermentable substrate.

The significant R:C ratio × YUFMP interaction for b and for cumulative gas at 48 and 96 h refines this interpretation. The stimulation was much larger in the 40:60 substrate, where the unsupplemented control (T1) had the lowest gas production of all treatments, than in the 60:40 substrate, where even the unsupplemented control (T5) produced 101.55 mL/0.5 g DM. The low gas production of T1 was not accompanied by a significantly lower total VFA concentration. Cumulative gas and VFA were however determined in separate sets of vials, VFA on only two vials per treatment and time point, and cumulative gas included indirect CO2 released from the bicarbonate buffer in addition to direct fermentation gas [52]. Moreover, one T1 vessel was excluded from the gas dataset (Section 2.9), so the T1 gas mean was based on two vessels. Consistent with its low gas production, T1 had the numerically lowest total VFA concentration at 12 h (44.11 mmol/L), although this difference was not significant. The T1 gas values should nevertheless be interpreted with caution. YUFMP was therefore most valuable where the basal substrate was least able to sustain fermentation, and its marginal benefit diminished when the substrate was already rich in fermentable material. Kanakai et al. [25] and Dagaew et al. [23], using structurally identical factorials of a yeast-fermented by-product across R:C ratios, reported the same dependence of supplement response on basal diet composition, and Phesatcha et al. [27] and Elghandour et al. [22] similarly found that the fermentative benefit of yeast-based additives is conditioned by the fibre-to-concentrate balance of the substrate.

### 4.3. Nutrient Degradability

The dominant determinant of degradability was the R:C ratio rather than YUFMP inclusion, with the 40:60 substrate exceeding the 60:40 substrate by approximately 7 percentage units for IVDMD and 9 units for IVTD at 24 h. This is the expected consequence of replacing rice straw (75.95% NDF, 43.50% ADF) with concentrate (43.15% NDF), and agrees with Phesatcha et al. [27], Kanakai et al. [25] and Zhang et al. [53], all of whom reported higher in vitro degradability and gas production at lower roughage proportions. The relevant observation for the present objective is that YUFMP inclusion did not compromise this advantage.

The effect of YUFMP on degradability depended on both incubation time and substrate fraction, and these two patterns should be considered separately. At 12 h, the interaction was significant for all four variables, and within the 40:60 ratio the unsupplemented T1 had the highest IVDMD. At this early stage, YUFMP contributed additional substrate that had not yet been degraded, which mathematically depresses the apparent degradability coefficient. By 24 h, this transient effect had resolved and the fibre-related fractions responded positively: IVNDFD and IVTD were highest at 6% YUFMP (47.90 and 69.30%) with significant linear contrasts, whereas IVDMD was highest at 2% and declined at 6%. The divergence between IVDMD and IVNDFD at the highest inclusion level is biologically coherent: phenolic compounds bind preferentially to soluble protein and readily available substrate, so a larger proportion of the degradation that does occur is fibre-derived [14,15]. Jalal et al. [12] similarly reported that mango by-products shifted fermentation without depressing fibre degradation, and Kanakai et al. [25] observed that the improved protein-to-fibre ratio at moderate inclusion was what drove their digestibility response. Nevertheless, the magnitude of these YUFMP effects on degradability (about 3 percentage units) was small relative to the R:C ratio effect.

### 4.4. Ruminal pH, Ammonia Nitrogen and Protozoal Population

Ruminal pH remained between 6.87 and 7.04 and was unaffected by treatment, confirming that the buffering capacity of the incubation medium was adequate and that neither the higher concentrate proportion nor YUFMP inclusion drove the system towards acidosis. These values lie well above the threshold of approximately 6.2, below which fibrolytic activity is impaired, so the degradability results cannot be attributed to a pH effect [25,27]. Yeast-derived mannan oligosaccharides and beta-glucans have been proposed to stabilise ruminal pH by supporting lactate-utilising bacteria [18,25], which is consistent with the stability observed here.

Ammonia nitrogen was likewise unaffected by treatment. Because yeast-fermented by-products have been reported to raise ruminal NH_3_-N owing to their high protein and non-protein nitrogen content [19,25], the nitrogen load carried by YUFMP needs to be quantified before this result is interpreted. On the basis of the composition in Table 1, the basal substrates supplied approximately 9.0 (40:60) and 7.3 (60:40) mg N per vial, and YUFMP at 6% added about 0.8 mg N, an increase of 9–11%. Even if this nitrogen had been released completely as ammonia, NH_3_-N in the 40 mL medium would have risen by at most about 2 mg/dL, a difference comparable to the SEM of the NH_3_-N data (0.60 and 1.10 mg/dL at 12 and 24 h, respectively). The absence of a detectable NH_3_-N response is therefore expected from the nitrogen balance alone and does not in itself demonstrate reduced deamination. Similarly, the urea entering the incubation amounted to only 0.03–0.09% of total substrate DM (0.3–0.9 g/kg DM), an order of magnitude below practical feeding rates. The concurrent increase in gas production is compatible with incorporation of the added nitrogen into microbial protein, and with the hypothesis that phenolic-protein interaction moderates nitrogen release [14,15], but neither can be confirmed without direct measurement of microbial protein synthesis and nitrogen partitioning. All NH_3_-N values remained above the 5 mg/dL generally regarded as the minimum for unrestricted microbial protein synthesis, so nitrogen was not limiting in any treatment [54].

The protozoal population declined linearly with YUFMP inclusion at both time points, reaching a 29.4% reduction at 6% YUFMP at 24 h. Rumen ciliates host a substantial fraction of the methanogen population as ecto- and endosymbionts, and their removal reduces interspecies hydrogen transfer to methanogens. Newbold et al. [55] reported an average 11% decrease in CH_4_ with complete defaunation, together with a 30% increase in microbial protein flow, and Guyader et al. [56] derived a quantitative positive relationship between protozoal concentration and CH_4_ emission across studies. A partial reduction of the magnitude observed here would therefore be expected to lower CH_4_ production modestly rather than substantially, which is consistent with the non-significant decline in CH_4_ volume discussed in Section 4.6. Across treatments, protozoal numbers at 24 h were closely correlated with CH_4_ concentration (r = 0.88, *p* = 0.004), but only weakly with CH_4_ volume (r = 0.44, *p* = 0.28), indicating that the decline in protozoa was associated more closely with the change in gas composition than with the total quantity of CH_4_ produced. The antiprotozoal response is plausibly associated with the phenolic fraction of the mango peel, which can disrupt protozoal membranes [14,15], and Kanakai et al. [25] and Polyorach et al. [19] found no protozoal response to yeast-fermented citric waste or cassava. Because only the final product was incubated, however, the present design cannot separate the contribution of the mango peel phenolics from that of the yeast waste, the urea or the additional substrate, and this attribution should be regarded as a working hypothesis.

### 4.5. Volatile Fatty Acid Profile

The R:C ratio governed the VFA profile at 24 h in the expected direction. The higher total VFA concentration of the 40:60 substrate (65.56 vs. 57.16 mmol/L) is consistent with its greater IVOMD and IVTD at the same time point, the higher acetate proportion of the 60:40 substrate reflects the larger contribution of structural carbohydrate from rice straw to the fermented pool, and the higher butyrate proportion at 40:60 is typical of concentrate-rich substrates in vitro [25,27,53]. Total VFAs did not respond significantly to YUFMP, even though cumulative gas at 24 h increased by up to 42%. VFAs were measured in vials separate from those used for gas production, and the numerical increase in total VFAs with YUFMP inclusion (from 60.40 to 62.55 mmol/L at 24 h) was small relative to its variance (SEM 3.43 mmol/L). A moderate stimulation of VFA production therefore cannot be excluded, but it was not large enough to be detected.

The more informative VFA result concerns the mechanism of the CH_4_ response. Propionate formation is the principal alternative sink for metabolic hydrogen in the rumen, so a reduction in methanogenesis achieved by redirecting hydrogen would be expected to raise the propionate proportion and lower the A:P ratio [7,52]. This is the pattern Jalal et al. [12] reported for mango by-products in vitro. It was not observed here, and across treatments the A:P ratio was not correlated with either CH_4_ concentration or CH_4_ volume at 12 or 24 h (|r| ≤ 0.52, *p* ≥ 0.19). YUFMP did not increase propionate at either time point and, at 12 h reduced it linearly, most markedly in the 60:40 substrate, while raising the A:P ratio. Notably, the 60:40 substrate was also the one in which CH_4_ concentration responded at the lowest dose (Section 4.6), the opposite of what a propionate-mediated hydrogen sink would produce. Without an alternative hydrogen sink in the VFA profile, total methanogenesis would not be expected to fall substantially, which is stoichiometrically consistent with the unchanged absolute CH_4_ volume (Section 4.6). The lower CH_4_ concentration is therefore best explained by a change in the composition of the fermentation gas, with CH_4_ diluted within a larger gas volume, rather than by a redirection of metabolic hydrogen. The transient reduction in propionate at 12 h, which had disappeared by 24 h, together with the tendency for butyrate to decline at 24 h, suggests that YUFMP altered the early course of fermentation rather than its end-point VFA profile.

### 4.6. Methane Concentration Versus Methane Volume

The CH_4_ concentration and CH_4_ volume data point in different directions, and reconciling them defines what the present results can support. Methane concentration in the headspace declined linearly and substantially with YUFMP inclusion at both 12 h (31.1% reduction from 0 to 6%) and 24 h (35.7%), yet neither absolute CH_4_ volume nor CH_4_ per gram of substrate DM differed significantly among treatments. The two findings are reconciled by the denominator: because YUFMP increased cumulative gas production by up to 42% at 24 h, a lower CH_4_ percentage of a larger gas volume yields a similar absolute quantity of CH_4_. The additional gas is likely to have consisted largely of CO_2_, derived both directly from fermentation and from the release of buffer CO_2_ by the acids produced [52], which diluted CH_4_ in the headspace. Consistent with this interpretation, CH_4_ concentration at 24 h was negatively correlated with cumulative gas production across treatments (r = −0.83, *p* = 0.011), whereas neither CH_4_ concentration nor CH_4_ volume was correlated with IVNDFD at either time point (*p* ≥ 0.19), indicating that the CH_4_ response was not driven by changes in fibre degradation. The primary effect of YUFMP was therefore to alter the composition of the fermentation gas rather than to reduce total methanogenesis, a reading supported by the absence of any shift towards propionate in the VFA profile (Section 4.5).

Protozoal suppression is best regarded as a secondary, potentially contributing mechanism. The numerically lowest CH_4_ volume at 24 h occurred at 6% YUFMP (5.70 mL, against 7.33 mL at 0%), and the linear contrast approached significance (*p* = 0.0810). The direction of this trend is consistent with the concurrent 29.4% decline in protozoa [55,56], but it was not statistically resolved. Statistical power for this variable was limited: CH_4_ volume carried the largest relative standard error in the dataset (SEM 0.93 mL against a mean of about 7 mL at 24 h), reflecting the inherent variability of headspace gas sampling. In addition, the metric of practical interest for a feed additive is CH_4_ per unit of degraded substrate rather than per incubation vial. Because degradability was maintained or improved while CH_4_ volume was unchanged, CH_4_ yield per unit of degraded substrate would be expected to decline. A modest protozoa-mediated reduction in methanogenesis therefore cannot be excluded, and comparable directional responses to phenolic-rich and yeast-based supplements have been reported in vitro [12,13,22]. Confirming it will however require greater replication and in vivo measurement of CH_4_ emission.

The significant R:C ratio × YUFMP interaction for CH_4_ concentration at 24 h adds a practical nuance. In the 40:60 substrate, CH_4_ concentration fell sharply only once YUFMP reached 4% (from 24.27 and 23.48% at 0 and 2% to 14.31 and 14.34% at 4 and 6%), whereas in the 60:40 substrate the reduction was already evident at 2% YUFMP (22.15% falling to 17.80%). At 2% YUFMP, the absolute CH_4_ volume was identical in the two substrates (8.00 mL in T2 and T6), whereas T6 produced more cumulative gas (41.67 vs. 36.20 mL/0.5 g DM) and had a lower protozoal count. The lower CH_4_ concentration of the 60:40 substrate therefore reflected greater dilution of a similar quantity of CH_4_. The higher butyrate proportion of the 40:60 substrate (16.15 vs. 11.72 mol/100 mol), a pathway that releases H_2_, may also have sustained a higher CH_4_ concentration at this ratio [52]. This suggests that a lower dose may be sufficient to alter CH_4_ concentration in the roughage-rich substrate, in which methanogenesis is in relative terms the more important hydrogen sink [7,52]. Because this observation rests on a single time point, it should be confirmed in further work.

The use of rice straw as the sole roughage may also have contributed to this interaction through the synchrony of ruminal energy and nitrogen supply. Rice straw is a slowly degraded, fibre-rich roughage (75.95% NDF; Table 1), and the 60:40 substrate consequently released fermentable energy more slowly than the 40:60 substrate, as reflected in its lower IVDMD and IVOMD at both incubation times (Table 2). YUFMP, in contrast, supplied nitrogen that is largely available early in fermentation, because approximately a quarter of its CP is non-protein nitrogen derived from urea (Section 4.1). Synchronising the rates of energy and nitrogen release can improve the efficiency of microbial protein synthesis [57,58], and a larger proportion of degraded substrate incorporated into microbial biomass would leave less substrate, and hence less metabolic hydrogen, available for VFA, CO_2_ and CH_4_ formation. The degree of synchrony could therefore have modified the CH_4_ response to YUFMP differently at the two R:C ratios. Several observations nevertheless suggest that any such effect was modest in the present study: NH_3_-N remained above 8.79 mg/dL in all treatments and was not affected by treatment, so nitrogen supply did not limit microbial growth at either ratio (Section 4.4), and the interaction was confined to CH_4_ concentration and did not extend to CH_4_ volume (*p* = 0.9459 at 24 h). Moreover, the benefits of nutrient synchrony demonstrated under controlled conditions have not been consistently reproduced in vivo [59]. Because the rates of carbohydrate and nitrogen release, the synchrony index and microbial protein synthesis were not measured, this mechanism cannot be confirmed with the present data, and it should be examined in future studies that compare roughages differing in degradation rate and measure microbial protein synthesis and CH_4_ production per unit of degraded substrate.

### 4.7. Practical Implications and Limitations

Taken together, the data identify 2–4% of substrate DM as the practical inclusion window for mango peel fermented with yeast waste. Within this range, gas production and fibre degradability were increased or maintained, protozoa and CH_4_ concentration were reduced, and rumen pH, NH_3_-N, and the 24 h VFA profile were unperturbed. At 6%, the gas and IVDMD responses had begun to plateau or reverse while the antiprotozoal and CH_4_-concentration effects continued to strengthen. The CH_4_-concentration response at 6% does not by itself justify the higher level, because it was not accompanied by a significant reduction in CH_4_ volume. This window coincides closely with the 4% upper limit identified by Kanakai et al. [25] for the analogous citric waste product, which strengthens the case that moderate inclusion is the general rule for this class of yeast-fermented by-product [5,23,25]. At these inclusion levels, the quantity of mango peel used per animal is modest. Because mango peel contributed approximately 77% of the YUFMP DM, a 300 kg growing animal consuming 2.5% of body weight as DM (7.5 kg DM/d) and receiving YUFMP at 4% of dietary DM would consume about 0.30 kg of YUFMP DM per day, corresponding to approximately 0.23 kg of peel DM per day, or about 85 kg of peel DM per animal per year. A 100-head feedlot would therefore utilise about 8.5 t of peel DM annually, together with the associated yeast waste. The waste-management benefit of YUFMP therefore depends on adoption across many farms rather than on high inclusion in individual diets, and its principal value lies in converting two perishable residues into a stable, protein-enriched supplement that can be stored and transported.

Several limitations qualify these conclusions. First, only the final product was incubated, and it was top-dressed rather than substituted. This design reflects the intended use of YUFMP, and the magnitude of the gas response indicates that it was not driven by the additional substrate mass (Section 4.2). It does not, however, allow the effects of the mango peel, the yeast waste and the urea to be separated, and a comparison of MP, YFMP and YUFMP at equal DM will be required to attribute the responses to individual components. Second, three gas-production vessels were excluded before analysis, producing unequal replication for the gas variables, as indicated in the footnote to Table 5. In addition, the 50 mL vials provided a limited headspace, so gas pressure may have risen transiently during the longer intervals between readings. Because all treatments were handled identically, this would have affected absolute values rather than treatment comparisons [43]. Third, in vitro systems lack the absorption and passage dynamics of the intact rumen, so the accumulation of fermentation end-products may exaggerate the inhibitory effects observed at the highest inclusion level [25,43]. Validation in an in vivo trial measuring intake, total-tract digestibility, nitrogen balance and CH_4_ emission is the necessary next step [24]. Fourth, the fermentation products of YUFMP itself (organic acids, ethanol and other volatile compounds) were not determined, and drying at 60 °C may have volatilised part of these compounds before incubation. The in vitro responses therefore refer to the dried product, which is also the form in which YUFMP would be stored and fed. Moreover, the fresh weight of the product after fermentation was not recorded, so DM loss could only be estimated (Section 4.1) and OM loss could not be determined, and because the peel was taken from a single pooled consignment, batch-to-batch variation in residual flesh, cultivar and fruit maturity, which may influence the composition of the product, remains to be assessed. Fifth, phenolic compounds were quantified per gram of dried extract, and because the extract yield was not recorded, the phenolic dose supplied to the incubation vials cannot be expressed on a feed DM basis. The attribution of the antiprotozoal response to peel phenolics therefore remains a hypothesis. Finally, microbial analysis was limited to total protozoa, so effects on methanogens and on the bacterial groups involved in fibre degradation and hydrogen metabolism can only be inferred indirectly from the fermentation end-products. These groups should be quantified by qPCR or 16S rRNA gene sequencing in future in vivo work.

## 5. Conclusions

Co-fermentation with fresh ethanol-industry yeast waste raised the crude protein content of mango peel from 4.39% to 17.00% DM, converting two discarded residues into a single nitrogen-bearing supplement that retained a substantial phenolic fraction. Including this product at 2–6% of substrate DM increased asymptotic and cumulative gas production by considerably more than the added substrate mass, maintained or improved fibre degradability, and linearly reduced both the protozoal population and the methane concentration of the fermentation gas without disturbing ruminal pH, ammonia nitrogen or the VFA profile at 24 h. Absolute methane volume was not significantly reduced and fermentation did not shift towards propionate. The lower methane concentration therefore reflects mainly a change in fermentation gas composition, with protozoal suppression as a possible secondary contributor, rather than confirmed methane abatement. An inclusion level of 2–4% of substrate DM is recommended for further evaluation. An in vivo study measuring intake, total-tract digestibility, nitrogen balance and methane emission, together with a comparison of the untreated and fermented peel products, is the appropriate next step before practical feeding recommendations are issued.

## Figures and Tables

**Table 1:** Ingredient composition of the concentrate and chemical and phytochemical composition of the concentrate, mango peel products, and rice straw.

Item	Concentrate	MP	YFMP	YUFMP	Rice Straw
Ingredients, % DM					
Cassava chips	45.00	-	-	-	-
Ground corn	10.00	-	-	-	-
Rice bran	10.00	-	-	-	-
Soybean meal	11.00	-	-	-	-
Brewers’ grains	11.00	-	-	-	-
Palm kernel meal	10.00	-	-	-	-
Molasses	0.50	-	-	-	-
Urea	0.50	-	-	-	-
Salt	1.00	-	-	-	-
Vitamin–mineral premix ^1^	1.00	-	-	-	-
Chemical composition					
Dry matter, %	93.02	92.54	92.69	92.32	92.85
Organic matter, % DM	93.81	94.15	88.78	89.44	85.38
Ash, % DM	6.20	5.85	11.22	10.56	14.63
Crude protein, % DM	15.53	4.39	13.20	17.00	4.81
Neutral detergent fibre, % DM	43.15	38.01	44.76	44.02	75.95
Acid detergent fibre, % DM	16.97	13.72	22.69	23.56	43.50
TPC (mg GAE/g extract)	-	100.30	75.73	73.49	-
TFC (mg QE/g extract)	-	110.14	120.38	120.85	-
DPPH (%)	-	35.72	37.51	40.52	-
ABTS (%)	-	43.78	29.29	25.26	-

MP = untreated mango peel; YFMP = mango peel fermented with yeast waste; YUFMP = mango peel fermented with yeast waste and urea. Only YUFMP was used in the in vitro treatments. Values are means of three analytical replicates (*n* = 3). ^1^ Mineral and vitamin quantities per kg of premix: vitamin A, 10,000,000 IU; vitamin D, 1,600,000 IU; vitamin E, 70,000 IU; Fe, 50 g; Zn, 40 g; Mn, 40 g; Cu, 10 g; I, 0.5 g; Co, 0.1 g; Se, 0.1 g. DM = dry matter; TPC = total phenolic content; TFC = total flavonoid content; DPPH = 2,2-diphenyl-1-picrylhydrazyl radical scavenging activity; ABTS = 2,2-azino-bis (3-ethylbenzothiazoline-6-sulfonic acid) radical scavenging activity; GAE = gallic acid equivalent; QE = quercetin equivalent.

**Table 2:** Effects of roughage-to-concentrate ratio and YUFMP inclusion level on in vitro nutrient degradability after 12 and 24 h of incubation.

Treatment	R:C Ratio	YUFMP (%)	IVDMD (%)		IVOMD (%)		IVNDFD (%)		IVTD (%)	
			12 h	24 h	12 h	24 h	12 h	24 h	12 h	24 h
T1	40:60	0	45.59 ^a^	60.64	46.58 ^a^	64.17 ^a^	36.23 ^a^	49.02	64.11 ^a^	71.31
T2	40:60	2	42.30 ^bc^	60.99	43.88 ^bc^	63.81 ^a^	32.42 ^b^	50.50	62.14 ^b^	72.27
T3	40:60	4	41.80 ^bc^	60.62	43.21 ^bc^	64.52 ^a^	34.45 ^a^	49.17	63.42 ^a^	71.64
T4	40:60	6	44.04 ^ab^	60.23	45.37 ^ab^	63.92 ^a^	34.52 ^a^	52.35	63.60 ^a^	73.52
T5	60:40	0	34.74 ^e^	51.76	36.20 ^e^	55.31 ^c^	24.77 ^d^	40.88	52.75 ^e^	62.87
T6	60:40	2	33.65 ^e^	56.25	35.27 ^e^	59.94 ^b^	26.16 ^d^	39.74	53.91 ^d^	62.40
T7	60:40	4	37.15 ^d^	55.47	38.87 ^d^	59.16 ^b^	26.11 ^d^	39.11	54.14 ^d^	62.21
T8	60:40	6	40.30 ^c^	51.56	41.49 ^c^	54.91 ^c^	29.36 ^c^	43.45	56.39 ^c^	65.09
SEM			0.71	0.84	0.74	0.80	0.56	0.99	0.32	0.58
Main effects										
R:C ratio	40:60		43.43	60.62 ^a^	44.76	64.11	34.41	50.26 ^a^	63.32	72.19 ^a^
	60:40		36.46	53.76 ^b^	37.96	57.33	26.60	40.80 ^b^	54.30	63.14 ^b^
YUFMP (%)		0	40.17	56.20 ^BC^	41.39	59.74	30.50	44.95 ^B^	58.43	67.09 ^B^
		2	37.98	58.62 ^A^	39.58	61.88	29.29	45.12 ^B^	58.02	67.33 ^B^
		4	39.47	58.05 ^AB^	41.04	61.84	30.28	44.14 ^B^	58.78	66.92 ^B^
		6	42.17	55.90 ^C^	43.43	59.42	31.94	47.90 ^A^	60.00	69.30 ^A^
Significance of main effects, interaction and polynomial trends										
R:C ratio			<0.01	<0.01	<0.01	<0.01	<0.01	<0.01	<0.01	<0.01
YUFMP level			<0.01	<0.05	<0.01	<0.05	<0.01	<0.05	<0.01	<0.05
R:C ratio × YUFMP			<0.01	0.0706	<0.01	<0.05	<0.01	0.5769	<0.01	0.5430
Linear			<0.05	0.5931	<0.05	0.7032	<0.05	<0.05	<0.01	<0.01
Quadratic			<0.01	<0.01	<0.01	<0.01	<0.01	<0.05	<0.01	<0.05

T1–T4: R40C60 with 0, 2%, 4%, and 6% YUFMP; T5–T8: R60C40 with 0, 2%, 4%, and 6% YUFMP. Superscripts are explained in Section 2.9; means within a column with different superscripts differ (*p* < 0.05). IVDMD = in vitro dry matter degradability; IVOMD = in vitro organic matter degradability; IVNDFD = in vitro neutral detergent fibre degradability; IVTD = in vitro true degradability; SEM = standard error of the mean.

**Table 3:** Effects of roughage-to-concentrate ratio and YUFMP inclusion level on fermentation characteristics and protozoal population after 12 and 24 h of incubation.

Treatment	R:C Ratio	YUFMP (%)	pH		NH_3_-N (mg/dL)		Protozoa (×10^5^ cells/mL)	
			12 h	24 h	12 h	24 h	12 h	24 h
T1	40:60	0	7.04	6.97	13.23	11.32	13.50	13.00
T2	40:60	2	7.01	6.88	12.48	9.28	10.50	11.50
T3	40:60	4	7.03	6.94	12.52	11.58	11.00	10.00
T4	40:60	6	7.02	6.89	11.15	10.02	9.00	9.50
T5	60:40	0	7.01	6.92	12.54	8.79	11.50	12.50
T6	60:40	2	7.02	6.87	12.73	8.88	11.00	9.50
T7	60:40	4	7.00	6.95	13.12	10.90	11.50	10.00
T8	60:40	6	7.01	7.02	12.57	11.98	9.50	8.50
SEM			0.03	0.06	0.60	1.10	1.02	0.88
Main effects								
R:C ratio	40:60		7.03	6.92	12.35	10.55	11.00	11.00
	60:40		7.01	6.94	12.74	10.14	10.88	10.12
YUFMP (%)		0	7.02	6.94	12.89	10.05	12.50	12.75 ^A^
		2	7.02	6.88	12.61	9.08	10.75	10.50 ^B^
		4	7.02	6.94	12.82	11.24	11.25	10.00 ^B^
		6	7.02	6.95	11.86	11.00	9.25	9.00 ^B^
Significance of main effects, interaction and polynomial trends								
R:C ratio			0.5251	0.6773	0.3759	0.6108	0.8661	0.1991
YUFMP level			0.9922	0.5128	0.3602	0.2627	0.0693	<0.05
R:C ratio × YUFMP			0.9203	0.4296	0.4170	0.3108	0.5485	0.7136
Linear			0.8473	0.5932	0.1699	0.1891	<0.05	<0.01
Quadratic			0.8535	0.3579	0.4458	0.6497	0.8661	0.3466

T1–T4: R40C60 with 0, 2%, 4%, and 6% YUFMP; T5–T8: R60C40 with 0, 2%, 4%, and 6% YUFMP. Superscripts are explained in Section 2.9; means within a column with different superscripts differ (*p* < 0.05). NH_3_-N = ammonia nitrogen; SEM = standard error of the mean. Protozoa are expressed as × 10^5^ cells/mL.

**Table 4:** Effects of roughage-to-concentrate ratio and YUFMP inclusion level on methane concentration and production after 12 and 24 h of incubation.

Treatment	R:C Ratio	YUFMP (%)	CH_4_ (%)		CH_4_ (mL)		CH_4_ (mL/g Basal DM)	
			12 h	24 h	12 h	24 h	12 h	24 h
T1	40:60	0	21.59	24.27 ^a^	2.62	6.86	5.25	13.72
T2	40:60	2	20.65	23.48 ^a^	4.20	8.00	8.39	16.00
T3	40:60	4	16.19	14.31 ^b^	4.39	6.83	8.79	13.66
T4	40:60	6	16.93	14.34 ^b^	3.10	5.25	6.20	10.51
T5	60:40	0	16.39	22.15 ^a^	2.94	7.81	5.87	15.62
T6	60:40	2	15.70	17.80 ^b^	3.81	8.00	7.62	16.00
T7	60:40	4	13.81	17.69 ^b^	2.87	7.17	5.74	14.33
T8	60:40	6	9.24	15.51 ^b^	2.18	6.15	4.36	12.30
SEM			1.89	1.26	0.77	0.93	1.53	1.87
Main effects								
R:C ratio	40:60		18.84 ^a^	19.10	3.58	6.74	7.16	13.47
	60:40		13.79 ^b^	18.29	2.95	7.28	5.90	14.56
YUFMP (%)		0	18.99 ^A^	23.21	2.78	7.33	5.56	14.67
		2	18.17 ^A^	20.64	4.00	8.00	8.01	16.00
		4	15.00 ^AB^	16.00	3.63	7.00	7.26	14.00
		6	13.08 ^B^	14.93	2.64	5.70	5.28	11.41
Significance of main effects, interaction and polynomial trends								
R:C ratio			<0.01	0.3883	0.2789	0.4335	0.2789	0.4335
YUFMP level			<0.05	<0.01	0.2921	0.1743	0.2921	0.1743
R:C ratio × YUFMP			0.5981	<0.05	0.6816	0.9459	0.6816	0.9459
Linear			<0.01	<0.01	0.7530	0.0810	0.7530	0.0810
Quadratic			0.6904	0.4278	0.0755	0.1765	0.0755	0.1765

T1–T4: R40C60 with 0, 2%, 4%, and 6% YUFMP; T5–T8: R60C40 with 0, 2%, 4%, and 6% YUFMP. Superscripts are explained in Section 2.9; means within a column with different superscripts differ (*p* < 0.05). CH_4_ = methane; DM = dry matter; SEM = standard error of the mean. CH_4_ volume was calculated for each vial from its CH_4_ concentration and the cumulative gas of its paired gas production vessel (Section 2.7); values therefore do not equal the product of the mean CH_4_ concentration and the mean cumulative gas production (Section 3.5). CH_4_ per gram is expressed on the basis of the basal substrate DM (0.5 g).

**Table 5:** Effects of roughage-to-concentrate ratio and YUFMP inclusion level on gas-production kinetics and cumulative gas production.

Treatment	R:C Ratio	YUFMP (%)	*b*	c	*L*	Cumulative Gas Production (mL/0.5 g DM)				
						6 h	12 h	24 h	48 h	96 h
T1	40:60	0	74.15 ^d^	0.0197	1.51	3.30	12.20	28.20	46.20 ^c^	61.30 ^c^
T2	40:60	2	92.27 ^c^	0.0236	1.44	5.13	21.07	36.20	64.53 ^b^	80.87 ^b^
T3	40:60	4	104.50 ^ab^	0.0273	1.43	6.40	26.80	48.00	79.10 ^a^	94.50 ^a^
T4	40:60	6	96.60 ^bc^	0.0240	1.69	4.37	19.57	38.77	68.90 ^ab^	84.03 ^ab^
T5	60:40	0	101.55 ^abc^	0.0203	1.82	4.30	16.90	34.90	65.80 ^b^	83.80 ^ab^
T6	60:40	2	109.47 ^a^	0.0224	1.52	5.00	23.20	41.67	74.10 ^ab^	93.70 ^a^
T7	60:40	4	100.67 ^abc^	0.0245	1.64	5.20	21.73	41.63	72.03 ^ab^	88.57 ^ab^
T8	60:40	6	104.33 ^ab^	0.0238	1.41	5.87	24.13	41.47	73.67 ^ab^	91.47 ^ab^
SEM			3.48	0.0020	0.24	1.05	2.88	3.95	4.14	4.11
Main effects										
R:C ratio	40:60		91.88	0.0236	1.52	4.80	19.91	37.79	64.68	80.17
	60:40		104.00	0.0227	1.60	5.09	21.49	39.92	71.40	89.38
YUFMP (%)		0	87.85	0.0200	1.67	3.80	14.55 ^B^	31.55 ^B^	56.00	72.55
		2	100.87	0.0230	1.48	5.07	22.13 ^A^	38.93 ^AB^	69.32	87.28
		4	102.58	0.0259	1.53	5.80	24.27 ^A^	44.82 ^A^	75.57	91.53
		6	100.47	0.0239	1.55	5.12	21.85 ^A^	40.12 ^A^	71.28	87.75
Significance of main effects, interaction and polynomial trends										
R:C ratio			<0.01	0.4934	0.5982	0.6655	0.3974	0.4076	<0.05	<0.01
YUFMP level			<0.01	0.0526	0.8702	0.2995	<0.05	<0.05	<0.01	<0.01
R:C ratio × YUFMP			<0.01	0.8062	0.5374	0.4889	0.2506	0.3060	<0.05	<0.05
Linear			<0.01	<0.05	0.6683	0.1438	<0.05	<0.05	<0.01	<0.01
Quadratic			<0.01	0.0660	0.5271	0.1631	<0.05	<0.05	<0.01	<0.01

T1–T4: R40C60 with 0, 2%, 4%, and 6% YUFMP; T5–T8: R60C40 with 0, 2%, 4%, and 6% YUFMP. Superscripts are explained in Section 2.9; means within a column with different superscripts differ (*p* < 0.05). *b* = asymptotic gas production (mL/0.5 g DM); c = fractional rate of gas production (h^−1^); *L* = lag time (h); DM = dry matter; SEM = standard error of the mean. Gas-production means are based on three vessels per treatment, except T1, T3 and T5 (two vessels each; Section 2.9).

**Table 6:** Effects of roughage-to-concentrate ratio and YUFMP inclusion level on total and individual volatile fatty acids and the acetate-to-propionate ratio after 12 and 24 h of incubation.

Treatment	R:C Ratio	YUFMP (%)	Total VFAs (mmol/L)		Acetate (mol/100 mol)		Propionate (mol/100 mol)		Butyrate (mol/100 mol)		A:P Ratio	
			12 h	24 h	12 h	24 h	12 h	24 h	12 h	24 h	12 h	24 h
T1	40:60	0	44.11	63.28	58.12	59.12	23.12 ^a^	24.30	18.75	16.57	2.52	2.43
T2	40:60	2	49.01	64.80	56.66	60.34	22.28 ^ab^	23.82	21.06	15.84	2.54	2.54
T3	40:60	4	47.87	68.41	56.88	58.22	23.14 ^a^	25.80	19.99	15.99	2.46	2.26
T4	40:60	6	50.63	65.73	57.86	59.88	21.56 ^abc^	23.93	20.57	16.19	2.69	2.50
T5	60:40	0	47.72	57.52	55.62	62.74	23.13 ^a^	23.43	21.25	13.82	2.41	2.69
T6	60:40	2	47.52	58.20	56.69	63.22	22.38 ^ab^	24.66	20.94	12.12	2.54	2.59
T7	60:40	4	45.56	56.68	57.82	63.22	20.35 ^c^	26.15	21.83	10.63	2.85	2.42
T8	60:40	6	47.48	56.24	57.97	63.32	21.32 ^bc^	26.39	20.71	10.29	2.72	2.40
SEM			1.71	3.43	1.04	0.84	0.48	1.06	0.87	1.09	0.09	0.13
Main effects												
R:C ratio	40:60		47.90	65.56 ^a^	57.38	59.39 ^b^	22.53	24.46	20.09	16.15 ^a^	2.55	2.44
R:C ratio	60:40		47.07	57.16 ^b^	57.02	63.12 ^a^	21.79	25.16	21.18	11.72 ^b^	2.63	2.52
YUFMP (%)		0	45.91	60.40	56.87	60.94	23.13	23.86	20.00	15.20	2.46	2.56
YUFMP (%)		2	48.26	61.49	56.68	61.78	22.33	24.24	21.00	13.98	2.54	2.57
YUFMP (%)		4	46.72	62.55	57.35	60.72	21.75	25.97	20.91	13.31	2.65	2.34
YUFMP (%)		6	49.05	60.98	57.91	61.60	21.44	25.16	20.64	13.24	2.70	2.45
Significance of main effects, interaction and polynomial trends												
R:C ratio			0.5094	<0.01	0.6420	<0.01	0.0609	0.3803	0.1140	<0.01	0.2518	0.3582
YUFMP level			0.3130	0.9328	0.6505	0.5536	<0.05	0.2671	0.6695	0.3189	0.0971	0.3025
R:C ratio × YUFMP			0.2686	0.8125	0.4294	0.6475	<0.05	0.5105	0.4071	0.4889	0.1024	0.5577
Linear			0.1834	0.8025	0.2779	0.7331	<0.01	0.1339	0.5241	0.0955	<0.05	0.2116
Quadratic			0.9984	0.5992	0.6190	0.9756	0.4854	0.4510	0.3334	0.4789	0.8171	0.5747

T1–T4: R40C60 with 0, 2%, 4%, and 6% YUFMP; T5–T8: R60C40 with 0, 2%, 4%, and 6% YUFMP. Superscripts are explained in Section 2.9; means within a column with different superscripts differ (*p* < 0.05). Total VFAs = total volatile fatty acids (mmol/L); acetate, propionate and butyrate are expressed as mol/100 mol; A:P = acetate-to-propionate ratio; YUFMP = mango peel co-fermented with yeast waste and urea; SEM = standard error of the mean.

## Data Availability

The data presented in this study are available on request from the corresponding author.

## Supplementary Materials

The following supporting information can be downloaded at https://www.mdpi.com/article/10.3390/ani16203237/s1. Table S1: Calculated chemical composition of the incubated substrates (basal substrate plus top-dressed YUFMP); Table S2: Pearson correlation coefficients (*r*) and p-values between CH4 and selected fermentation variables across treatment means (*n* = 8) after 12 and 24 h of incubation.
