# Supplementary material for: Mango Peel Fermented with Ethanol-Industry Yeast Waste and Urea Modulates In Vitro Rumen Fermentation, Nutrient Degradability, and Methane Concentration
Source: Animals (Basel). 2026 Oct 15;16(20):3237. doi: 10.3390/ani16203237 (PMC13700581; doi:10.3390/ani16203237)
Supplement: Supplementary file 1 [file animals-16-03237-s001.zip › animals-4633634-supplementary.pdf]

Table S1. Calculated chemical composition of the incubated substrates (basal substrate plus top-dressed YUFMP).

Treatment	R:C ratio	YUFMP (% of basal DM)	YUFMP added (g DM/vial)	Total DM incubated (g)	OM (% DM)	Ash (% DM)	CP (% DM)	NDF (% DM)	ADF (% DM)	N supplied (mg/vial)
T1	40:60	0	0.00	0.50	90.44	9.57	11.24	56.27	27.58	8.99
T2	40:60	2	0.01	0.51	90.42	9.59	11.35	56.03	27.50	9.27
T3	40:60	4	0.02	0.52	90.40	9.61	11.46	55.80	27.43	9.54
T4	40:60	6	0.03	0.53	90.38	9.63	11.57	55.58	27.35	9.81
T5	60:40	0	0.00	0.50	88.75	11.26	9.10	62.83	32.89	7.28
T6	60:40	2	0.01	0.51	88.77	11.24	9.25	62.46	32.71	7.55
T7	60:40	4	0.02	0.52	88.78	11.23	9.40	62.11	32.53	7.82
T8	60:40	6	0.03	0.53	88.79	11.22	9.55	61.77	32.36	8.09

T1–T4: R:C 40:60 (0.20 g rice straw + 0.30 g concentrate DM per vial) with 0, 2%, 4%, and 6% YUFMP; T5–T8: R:C 60:40 (0.30 g rice straw + 0.20 g concentrate DM per vial) with 0, 2%, 4%, and 6% YUFMP.

Values were calculated as the DM-weighted means of the analysed composition of rice straw, concentrate and YUFMP (Table 1) and the amounts of each component incubated; the mixed substrates were not analysed directly. N supplied = CP supplied / 6.25.

YUFMP = mango peel co-fermented with yeast waste and urea; R:C = roughage-to-concentrate ratio; DM = dry matter; OM = organic matter; CP = crude protein; NDF = neutral detergent fibre; ADF = acid detergent fibre; N = nitrogen.

Table S2. Pearson correlation coefficients (*r*) and *p*-values between CH_4 and selected fermentation variables across treatment means ($n = 8$) after 12 and 24 h of incubation.

Variable	CH_4 concentration (%)				CH_4 volume (mL)			
	12 h		24 h		12 h		24 h	
	r	*p*	*r*	*p*	*r*	*p*	*r*	*p*
IVNDFD	0.51	0.194	0.00	0.997	0.18	0.676	−0.47	0.237
Cumulative gas production	−0.60	0.116	**−0.83**	**0.011**	0.45	0.258	−0.12	0.780
Protozoal count	0.52	0.188	**0.88**	**0.004**	−0.06	0.880	0.44	0.277
A:P ratio	−0.52	0.190	0.49	0.220	−0.46	0.249	0.46	0.253

Correlations were computed with the CORR procedure of SAS on the eight treatment means (T1–T8), because CH_4 and the other variables were determined in separate vials (CH_4, protozoa and VFA in Set B; IVNDFD in Set C; cumulative gas production in Set A). Values in bold are significant ($p < 0.05$).

CH_4 = methane; IVNDFD = *in vitro* neutral detergent fibre degradability (%); cumulative gas production is expressed as mL/0.5 g DM at the corresponding time; protozoal count is expressed as $\times 10^5$ cells/mL; A:P = acetate-to-propionate ratio; DM = dry matter.
